# Conservatism in Abdominal and Pelvic Surgery

**Published:** 1894-09

**Authors:** J. C. Olmsted

**Affiliations:** Atlanta, Ga.


### CONSERVATISM IN ABDOMINAL AND PELVIC SURGERY.*

By J. C. OLMSTED, M.D.,
ATLANTA, GA.

The very wide scope of the subject set for discussion this evening has caused the speaker, as one of those requested to open the discussion, to limit his remarks chiefly to the subject of "Conservative Abdominal and Pelvic Surgery" in the *puerperal state;* and he has been led to take this line of thought from the consideration that, as *general practitioners,* we are *all* interested in it, and even yet, despite the inroads of specialism into the region of obstetric practice, have to face conditions and complications in this branch of our art, which, as long as we are permitted to follow it, requires that we should have clear and decided views in regard to its practical details. It might at first sight appear unnecessary, or at least a *truism,* to argue for "conservative surgery" in the condition mentioned, and to deprecate the *prompt* resort to *mechanical* and *surgical* procedures, to the exclusion of well selected and well proven medical remedies, were it not that we too frequently hear or read of the resort, in certain of these cases, to aggressive and, as I think, uncalled for and mischievous surgical measures, which but too frequently result in disaster to the patient and discredit to the medical profession. Far be it from me to underrate or decry the wonderful progress and magnificent achievements of modern surgery, especially in the regions which are under discussion. My conservatism is not of that kind or description, but it does seem to me that in our enthusiasm over strictly surgical methods, we may, and do at times, amidst the glamour of success, lose our equipoise, and become guilty of a narrow and mischievous specialism, to the exclusion of that matured, considerate and well rounded judgment, which must ever, in the achievement of success in our practice, take cognizance of differing conditions, and of all aspects of treatment, both medical and surgical. To illustrate: It has happened

* Read before the Atlanta Society of Medicine, June, 1894.

probably to every practitioner of any considerable experience to have observed, at times in his obstetrical practice, cases in which, a few days after a perfectly normal and successful labor, fever has supervened, and that, too, of marked character; temperature, it may be, of 103–103½° or 104° F.; lochial discharge considerably diminished, or scarcely noticeable, while symptoms of blood poisoning were absent, or present in a very subordinate degree. Such cases, while the source of considerable anxiety, and entailing increased watchfulness, have often been found to yield in a few days to mild medical treatment, leaving no evidence of local mischief of an inflammatory character in the pelvic structures. We have often been puzzled in such cases to account for this degree of fever in the absence of signs of local inflammation or other systemic symptoms. Probably this condition is somewhat analogous to what is known as "aseptic surgical fever," due to absorption of the products of tissue change at the seat of injury. Such is the explanation of Gengmer and Volkman of this condition as observed not infrequently in purely surgical cases, where every precaution of "listerism" has been faithfully observed, and it seems to have "no prognostic influence." I remember some years ago that my friend, Dr. W. P. Nicolson, gave me the particulars of a case in which he had performed an amputation, I think, at the lower third of the thigh, and in which the most rigid aseptic precautions had been observed. Two or three days after the operation, he was much concerned to find a very high rise of temperature, which caused him, although the patient had no other untoward symptoms, to remove the dressings and examine the wound. He found everything fresh and clean and redressed the wound. The cure progressed to a rapid recovery, with no other disturbing conditions, and without the formation of any pus at all. Returning to the form of fever in the puerperal state, which I have mentioned, Lusk well says: "While in puerperal women we can never exclude the possibility of the septic infection of puerperal wounds, it is in accordance with clinical experience to assume that a high fever, belonging to the aseptic class, may coincide with a septic process of insignificant proportions."

Such has been the experience of the speaker, and I doubt not that there are those here who will bear him out in it, and also that they have *cured* such cases *without* recourse to the numerous "curettings," whether with the "blunt" or "sharp curette," which seems to be in danger of becoming as *fashionable* as *laparotomy* for *puerperal peritonitis!* I do not wish to be misunderstood. There doubtless *are* cases in which the curette is indicated to remove decomposing decidual membrane or placental débris; but where this condition is assumed to exist, it is desirable, I think, to first try the effect of antiseptic douches, vaginal and intrauterine, before resorting to the curette; for it is not to be forgotten, that the scraping of this surface, especially if repeated, *opens* up and *exposes* a large *absorbent surface,* and may then insure the very evil which it is endeavored to correct, and thus invite the enemy in through many open gates! The speaker has heard of a curetting of this sort, where *all* that was obtained was a *small normal* blood *clot,* which probably was scraped from the mouth of some small sinus where it *belonged.* Again, while not proposing to enter upon the great field of puerperal fever, with its many and varied *local* manifestations, and differing forms, I would like to say of that form characterized by *general peritonitis,* that up to the present date it would appear that the strictly *medical* treatment is yet our main stay and hope. I must confess that I yet entertain a profound respect for the opium plan of treatment as advocated by Alonzo Clark, Barker and others in the past, and more recently by Lusk and others. I also have confidence in quinine, phenacetine and sodium salicylate as antipyretics in this disease, and cold by the wet pack is of value, in the same connection. These and other allied remedies seem more rational to me than the more than doubtful results of "*opening* the *peritoneum*" for *pus,* which may or may *not* exist there, and which, granting that it does exist, does not preclude the eminent probability of the subperitoneal and other tissues being *already* infiltrated with the special bacteria, upon the presence of which I believe that the general consensus of modern medical opinion ascribes the general septic symptoms observed in such cases.

The speaker must confess that he has not been able to get access to any published statistics upon the subject of *laparotomy* as performed for *puerperal peritonitis*, and while he holds himself ever open to conviction and new light, cannot but deprecate the retiring modesty of those who, while advocating this radical procedure, fail to furnish the evidence justifying it; and it is greatly to be apprehended that the collection of the statistics is left for the undertaker!

Of course, in the foregoing remarks, no reference is intended to the opening and closing of such localized and extraperitoneal abscesses as occur at times as the result of certain inflammatory diseases which may complicate the puerperal state. The remarks are offered in all frankness and with, I trust, all the humility which should characterize "the general practitioner."

---

